# Effectiveness and Safety of Mycophenolate Mophetil in Myasthenia Gravis: A Real-Life Multicenter Experience

**DOI:** 10.3390/brainsci14080774

**Published:** 2024-07-31

**Authors:** Claudia Vinciguerra, Anna D’Amico, Liliana Bevilacqua, Nicasio Rini, Maria D’Apolito, Eliana Liberatoscioli, Roberto Monastero, Paolo Barone, Filippo Brighina, Antonio Di Muzio, Vincenzo Di Stefano

**Affiliations:** 1 Neurology Unit, Department of Medicine, Surgery and Dentistry “Scuola Medica Salernitana”, University Hospital San Giovanni di Dio e Ruggi D’Aragona, 84131 Salerno, Italy; 2 Department of Biomedicine, Neuroscience and Advanced Diagnostics (BIND), University of Palermo, 90127 Palermo, Italyvincenzo19689@gmail.com (V.D.S.); 3 Department of Neuroscience, Imaging and Clinical Sciences, “G. d’Annunzio” University, 66100 Chieti, Italy

**Keywords:** myasthenia gravis (MG), mycophenolate mofetil (MMF), autoimmune disease, neuromuscular junction, immunosuppressive therapy, symptom control

## Abstract

Background: Myasthenia gravis (MG) is an autoimmune disease characterized by fluctuating muscle weakness due to autoantibodies targeting neuromuscular junction proteins. Mycophenolate mofetil (MMF), an immunosuppressive therapy, has shown potential for managing MG with fewer side effects compared to other treatments. This study aims to evaluate the effectiveness and safety of MMF in MG patients in a real-life multicenter setting. Methods: A retrospective cohort study was conducted on generalized MG patients, refractory to azathioprine (AZA) and treated with MMF alone or with steroids, at three Italian centers from January 2011 to February 2024. Patients were assessed using the Myasthenia Gravis Foundation of America (MGFA) classification, MG composite score (MGCS), and MG activity of daily living (MGADL) scores at baseline, 6, 12, 18, and 24 months. Statistical analyses included the Spearman correlation, the Friedman test, and ANOVA. Results: Thirty-two patients were enrolled (13 males, mean age 66.5 ± 11.5 years). Significant improvements in MGADL and MGCS scores were observed at 6 and 12 months (*p* < 0.001), with continued improvement over 24 months. Side effects were reported in 12% of patients. MMF showed a faster onset of symptom control compared to azathioprine, with a significant improvement noted within 6 months. Conclusions: A recent study found that MMF and AZA were equally effective in improving patients’ quality of life, but because AZA had more serious adverse events than MMF, lower doses of AZA were therefore recommended to reduce the adverse events while maintaining efficacy. Conversely, results showed that MMF is effective and well-tolerated in the long-term management of MG, providing faster symptom control and a favorable safety profile. Future prospective studies with larger cohorts are needed to confirm these findings and explore sex differences in response to MMF treatment.

## 1. Introduction

Myasthenia gravis (MG) is an autoimmune disease characterized by autoantibodies binding specific proteins involved in the neuromuscular junction function (NMJ) [1]. This mechanism disrupts the nerve–muscle transmission, causing fluctuating weakness and symptoms such as difficulty speaking and swallowing, ptosis, and double vision. In severe cases, patients may experience myasthenic crises (MG), marked by life-threatening respiratory complications due to weakened bulbar and diaphragmatic muscles [2]. The clinical presentation, treatment response, and disease mechanism differ among MG subgroups, which are classified according to the Abs pattern that includes anti-acetylcholine receptor (AChR), anti-muscle-specific kinase (MuSK) antibodies, low-density lipoprotein receptor-related protein 4 (LRP4) antibodies, and different clinical phenotypes (ocular or generalized) [3,4].

The long-term disease management consists of a stepwise therapeutic approach aimed at symptom control and minimizing adverse effects [5]. Treatment options include symptomatic therapies enhancing neuromuscular conduction and immunosuppressive therapies targeting autoimmune aggression at the NMJ [6]. Additionally, short-term immunotherapies are available for managing flare-ups or myasthenic crises. Individualized treatment choices are crucial for achieving satisfactory symptom management [3].

Corticosteroids are the primary immunosuppressive treatment, but other agents come into play in the case of non-response, or for the achievement of steroid-sparing effects, or to address severe corticosteroid side effects [3,5].

Mycophenolate mofetil (MMF) is a synthesized prodrug of mycophenolic acid (MPA) that inhibits the immune system by selectively depleting guanosine and deoxyguanosine in T- and B-lymphocytes. MMF presents a lower risk of serious organ toxicity and lower incidence of late-induced malignancies compared to other immunosuppressive therapies [7]. MPA, originally isolated from Penicillium glaucum, inhibits inosine monophosphate dehydrogenase (IMPDH) in the purine synthesis pathway, crucial for T- and B-lymphocyte proliferation [8]. After gastrointestinal absorption, MMF is converted to MPA, which undergoes glucuronidation and enterohepatic recycling, with a plasma concentration peak within 1–3 h and an elimination half-life of about 17 h. MMF is used to prevent organ transplant rejection and, due to its lower risk of serious organ toxicity and lower incidence of late-induced malignances compared to others immunosuppressive therapies, is also an attractive option for MG treatment [8,9].

The starting MMF dose is 500 mg to 1000 mg twice daily and most patients tolerate daily doses of 1000–2500 mg well. Three randomized trials suggest that MMF is ineffective when used for 12 and 36 weeks and leads only to mild improvement in neurophysiological measures such as single fiber EMG (SFEMG) parameters [10,11]. However, widespread clinical experience supports its efficacy in long-term MG management. In retrospective trials of patients treated with MMF, 50–75% of patients experienced improved symptoms after 5 to 12 months, and 50% achieved remission, with a low percentage of side effects [7,12,13]. In a study, after 24 months of combined MMF and corticosteroid treatment, 80% of the patients achieved a desirable outcome, as did patients who were on MMF monotherapy [13]. However, it is important to note that MMF is associated with certain risks, including teratogenicity and gastrointestinal effects, while it has fewer adverse effects on bone health, weight, cataract formation, and hypertension compared to corticosteroids. Teratogenicity is a significant concern, and women of childbearing potential should use effective contraception while on MMF and be advised about the potential risks to the fetus. Thus, several in vivo studies have supported the existence of a specific MMF embryopathy [14]. Physicians managing women on MMF therapy should be aware of the potential risk of this drug causing specific embryopathy and should ensure that treatment is discontinued at least six weeks before conception [14]. Gastrointestinal side effects, such as diarrhea, are also relatively common and can affect patient compliance with the treatment [9]. Typical dosing ranges from 1500 mg to 3000 mg per day, divided into two or three doses [15]. While plasma levels generally do not require monitoring, regular complete blood-cell counts are recommended due to the potential for leukopenia, anemia, pancytopenia, and agranulocytosis.

Despite numerous open-label studies and case series suggesting the effectiveness of MMF in treating myasthenia gravis (MG), the literature remains unclear, particularly regarding its speed of action, long-term efficacy, and safety profile [16]. Given the paucity of comprehensive studies addressing these aspects, our study aims to fill this gap. We present a multicenter retrospective real-life study designed to evaluate both the clinical improvement and the side effects experienced by a cohort of MG patients treated with MMF. By focusing on these key objectives, our research seeks to provide a more definitive understanding of MMF’s impact on MG management.

## 2. Materials and Methods

### 2.1. Study Population and Study Design

This retrospective cohort study was conducted for generalized MG patients treated with MMF who were followed at 3 Italian myasthenia gravis centers at the University Hospitals of Palermo, Salerno, and Chieti from January 2011 to February 2024. The diagnosis of MG was based on a clinical history characterized by fluctuating symptoms of fatigue and muscle weakness along with one or more positive results in neurophysiological tests such as repetitive nerve stimulation (RNS), single fiber electromyographic (SFEMG) study, or antibody research.

Specifically, positive RNS was defined as a decrease of greater than 10% between the first and fifth compound muscle action potential (CMAP) amplitudes in any of the nerves tested after 3–5 Hz stimulation or SFEMG positivity evaluated as an increase in jitter evaluated on at least 10 pairs associated or not with the presence of at least 3 blocks, and anti-AChR antibody (AChR-Ab) titers >0.45 nmol/L were defined as the presence or absence of anti-MuSK antibodies.

### 2.2. Clinical Measures

Each patient was classified using the Myasthenia Gravis Foundation of America (MGFA) classification [13], the antibody serotype, and the onset phenotype [17]. Furthermore, MG composite score (MGCS) [18] and MG activity day living (MGADL) [19] at baseline, 6, 12, 18, and 24 months were retrospectively collected for each patient. Both MGCS and MGADL are essential clinical tools for assessing MG severity and impact. MGCS evaluates clinical manifestations with scores ranging from 0 to 50, where higher scores indicate more severe damage. On the other hand, the MGADL scale is a patient-reported outcome measure that assesses the impact of MG on daily activities. Each item is scored on a scale from 0 (no impairment) to 3 (severe impairment), with a total score ranging from 0 to 24. Higher scores indicate greater disability. These tools enable us to quantitatively assess changes in disease severity and patient function, providing a comprehensive evaluation of treatment efficacy and safety.

Patients were excluded from the analysis if they met any of the following conditions: (1) thymectomy within the last 12 months; (2) inadequate response to MMF after at least 24 weeks of treatment; (3) a follow-up period of less than 6 months; (4) insufficient baseline data.

The evaluations at predefined follow-up times aimed to (a) identify the comorbidity and any side effects from MMF (hematological, hepatic, infectious, or neoplastic problems) requiring the adjustment of therapy or its suspension, or a switch or add-on to other drugs, and (b) note the reduction in ADL and MGCS scores at a single follow-up evaluation.

Patients experiencing clinical relapse triggered by factors such as infections, pregnancy, or the use of inappropriate drugs, or those evaluated less than 6 months after MMF reduction, were also excluded from the analysis.

### 2.3. Statistical Methods

Descriptive statistics were calculated for the demographic and clinical characteristics of the study population. Continuous variables (age, duration of the disease, and clinical scale scores) are presented as mean ± standard deviation (SD), while categorical variables (gender, MGFA classification, serotype, onset phenotype, thymectomy surgery, comorbidities, and treatments) are expressed as frequencies and percentages. The Spearman correlation was used to assess the relationship between the switch or add-on treatment with MMF and clinical outcomes, including MGADL and MG composite score (MGCS) at baseline, 6 months, and 12 months. The Friedman test for repeated measures was employed to evaluate the evolution of MGADL and MGCS over 24 months. The Wilcoxon test was used for pairwise comparisons between individual time points.

Efficacy analysis was carried out, evaluating the change in clinical scale scores at significant time points (6, 12). The variations in the scale were calculated using the following formulas:

$$\Delta 6_{months}=\frac{{Score}_{6months}-{Score}_{baseline}}{Score_{baseline}}$$

$$\Delta 12_{months}=\frac{{Score}_{12months}-{Score}_{baseline}}{Score_{baseline}}$$

These delta variations (Δ6_months_ and Δ12_months_) represent the relative change from baseline to 6 months and 12 months, respectively.

To understand the relationship between the delta variations and various clinical variables, we performed an analysis of variance (ANOVA). The clinical variables considered in the analysis included gender, clinical phenotype, serotype, thymectomy status, and MGFA classification at disease onset. The ANOVA was used to determine if there were statistically significant differences in the delta variations of the clinical scale scores across the different levels of these clinical variables. Statistical significance was set at *p* < 0.05.

Informed written consent was obtained from all subjects of the study, which was approved by the local ethics committee.

## 3. Results

### 3.1. Patient Demographics and Clinical Characteristics

A total of 32 patients were retrospectively enrolled (13 males, with a mean age of 66.5 ± 11.5 years and with a mean age at disease onset of 59.4 ± 12.8 years and a mean disease duration of 96.8 ± 68.5 months). MGFA classifications at disease onset were MGFA I (*n* = 7), MGFA IIA (*n* = 2), MGFA IIB (*n* = 9), MGFA IIIA (*n* = 8), MGFA IIIB (*n* = 5), and MGFA V (*n* = 1). Thymectomy was performed in eight patients (25%). Serotype distribution included acetylcholine receptor (AChR) antibodies in 26 patients (81%), muscle-specific kinase (MuSK) antibodies in two patients (6%), and double-seronegative in four patients (13%). At baseline, the mean MGADL score was 6.4 ± 5.3, and the mean MGCS was 7.6 ± 4.3 (see table). All patients had a generalized MG at the start of treatment with mycophenolate therapy (Table 1).

At 6 months, the mean MGADL and MGCS scores were 5.1 ± 0.8 and 6.2 ± 2.1, respectively. At 12 months, the mean MGADL score was 4.8 ± 1.8, and the mean MGCS score was 5.8 ± 2. Comorbidities included hypertension 15 (47%), osteoporosis 11 (35%), gastrointestinal diseases 9 (28%), thyroid disease 6 (19%), psychiatric disease (12%), and diabetes 3 (9%).

### 3.2. Treatment and Side Effects

**Figure 1:**
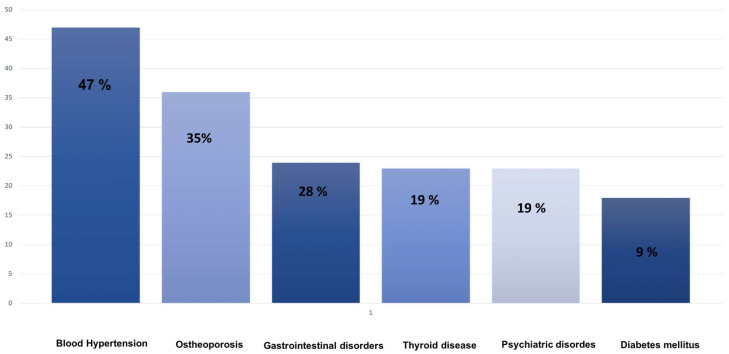
Bar diagram depicting the percentage distribution of comorbidities in the study population.

All patients had switched from azathioprine (AZA), at a medium dosage of 200 mg, to MMF after an average of 38 ± 18 months. Concomitant treatments included pyridostigmine (88%) and prednisone (88%) at the respective baseline dosage of 90 mg and 17.5 mg. Among the whole population, only four patients showed side effects (12.5%), scored as G1 according to Common Toxicity Criteria for Adverse Events, Version 5.0 (CTCAE, V5.0). Specifically, one patient showed G1 anemia, one patient showed G1 diarrhea, and two patients showed G1 elevation of liver enzymes (Figure 1). None presented any infections or neoplasms.

Four patients switched to another drug due to ineffectiveness, while in four others, a monoclonal antibody was added to MMF (Table 1).

### 3.3. Correlation Analysis and Clinical Improvements

**Figure 2:**
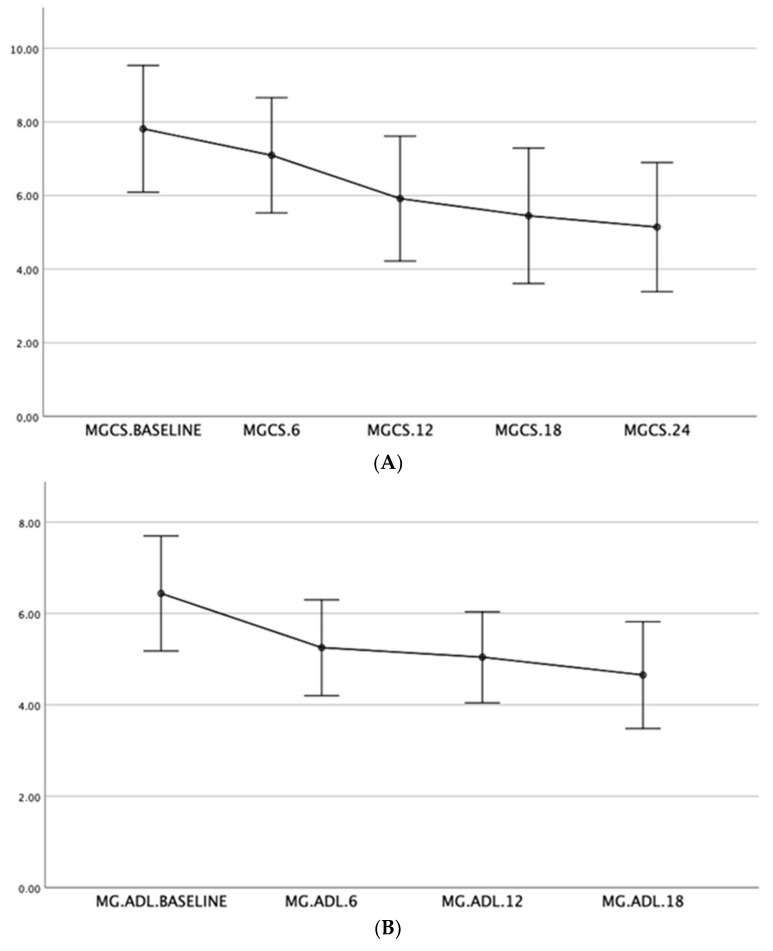
Illustrative graph of the trend improvement in the MGCS scale (**A**) and MGADL (**B**) at different time points (from baseline, 6, 12, 18, and 24 months).

Spearman correlation indicated that switching to MMF correlated with improvements in MGADL and MGCS at baseline, 6 months (*p* = 0.003, r = −0.48), and 12 months (*p* = 0.002, r = −0.53), along with side effect development and steroid dosage. Higher steroid doses correlated with a lower probability of switching to MMF (*p* = 0.003, r = −0.43). Thymectomy correlated with earlier disease onset, with thymectomy patients predominantly having early-onset MG (*p* = 0.001, r = −0.59). Significant improvements in MGADL were observed from baseline to 6 months (*p* = 0.001, r = 0.60) and from 6 to 12 months (*p* = 0.003, r = 0.47), with a reduction of two points. MGCS also significantly improved between baseline and 6 months (*p* = 0.001, r = 0.57), and from 6 to 12 months (*p* = 0.001, r = 0.61), with a reduction of three points. The Friedman test confirmed significant overall improvements in MGADL (*p* = 0.003) and MGCS (*p* < 0.001) over 24 months, with MGADL improving by two points and MGCS by three points. (Figure 2A,B).

**Figure 3:**
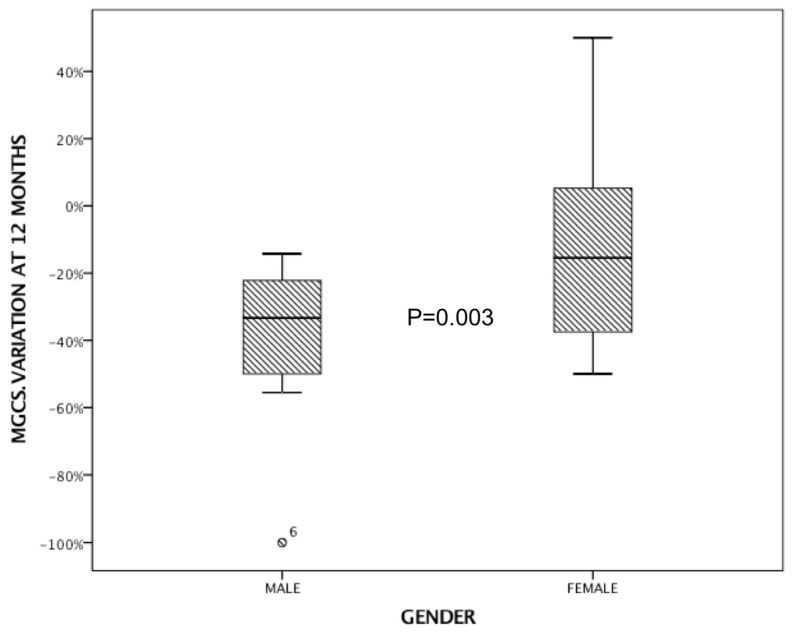
The ANOVA test showing a significant correlation between the delta variations in MGCS scores at 12 months and sex.

The ANOVA test revealed a significant correlation between the delta variations in MGCS scores at 12 months and sex. Specifically, this indicated that the change in MGCS scores over the 12-month period was significantly different between males and females (*p* = 0.003), with males responding better to MMF (Figure 3).

However, no significant correlations were found between the delta variations in MGCS or MGADL scores and other clinical variables, including serotype (AChR, MuSK, and double-seronegative *p* = 0.5), clinical phenotype (early or late onset *p* = 0.073), thymectomy status (*p* = 0.08), and MGFA classification at disease onset (MGFA I, IIA, IIB, IIIA, IIIB, V, *p* = 0.23). This suggests that these factors did not significantly influence the change in MGCS scores over the 12-month period.

**Figure 4:**
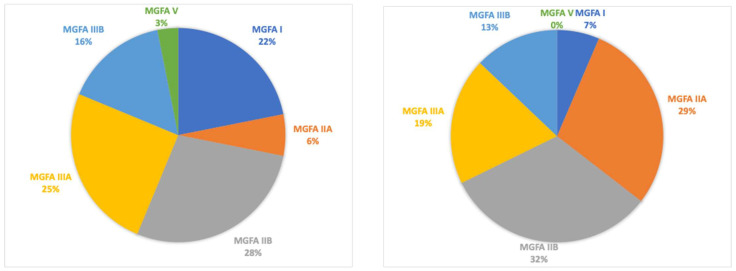
The distribution of MGFA classification among patients at the start of MMF (**left**) and at last follow-up (**right**).

Finally, MGFA scores at follow-up presented some differences: a reduction in patients with class VI and V was reported with an increased count in classes II and III (Figure 4).

## 4. Discussion

The findings from this retrospective study demonstrate significant improvements in MGADL and MGCS scores following the switch from AZA to MMF in patients with generalized MG and mild moderate disease activity. Significant improvements were observed at both 6 and 12 months post-switch, indicating the efficacy of MMF in managing MG symptoms.

Previous studies have reported similar findings regarding the efficacy of MMF in MG. For instance, Sanders et al. [11] conducted a randomized trial comparing MMF to a placebo and found that MMF was effective in reducing MG symptoms, as evidenced by improvements in quantitative MG (QMG) scores. Our study aligns with these findings, showing significant improvements in both MGADL and MGCS scores over 24 months, further supporting the use of MMF as an effective treatment option for MG. Interestingly, we observed a rapid improvement from the first 6 months with continuous amelioration until 24 months, with MGADL improving by two points and MGCS by three points (Figure 2). This quite rapid rate of onset when compared to AZA places MMF among drugs with a relatively faster onset of effect, allowing more rapid disease control. Another interesting insight comes from the comparison of the effect of MMF depending on the sex at birth. Indeed, a more pronounced effect on MGCS was demonstrated in males, with a reduction of over 30% (Figure 3). The starting MMF dose is 500 mg to 1000 mg twice daily, and the usual maintenance dose is 1000 to 1500 mg twice daily.

In terms of safety profile, our results showed that twelve percent of the patients experienced side effects, including anemia, diarrhea, and elevated liver enzymes, without infections or neoplasm. While these effects are typically less severe than those associated with other immunosuppressants, they can still impact the patient’s quality of life and may require dose adjustments or additional medications to manage symptoms. This is consistent with the previous literature, which has generally shown MMF to have a favorable safety profile compared to other immunosuppressants like AZA [12]. It is a well-tolerated medication, and the more common side effects reported include nausea, diarrhea, and infections including urinary tract infection and herpes zoster reactivation, and only rare cases of bacterial meningitis, viral encephalitis, and viral enteritis. Leukopenia can occur but rarely requires discontinuation [20,21].

A recent study compared the effectiveness of MMF with that of AZA and demonstrated that more than half of patients treated with both immunosuppressants experienced an improved quality of life, without differences in terms of clinical efficacy. Nonetheless, the adverse events associated with AZA were potentially more severe than those associated with MMF, although the latter resulted teratogenic [22]. The authors therefore advocated doses of AZA lower than those recommended as being equally effective, with a dose-dependent reduction in adverse events.

By contrast, our study showed that MMF can offer a faster alleviation of symptoms, with a significant improvement observed within 6 months from the switch. This is particularly relevant considering that AZA often requires a longer period to achieve therapeutic effects. Moreover, MMF was associated with fewer side effects, enhancing its safety profile compared to AZA. This rapid effectiveness may be crucial for patients who need immediate symptom control and for those who want to reduce their dependence on corticosteroids such as prednisone. Furthermore, we can speculate that the phenomenon of “higher steroid doses correlated with lower probability of switching to MMF” can be attributed to achieving maximum therapeutic efficacy with minimal adverse effects. Higher steroid doses may provide more effective immunosuppression, leading to better control of disease symptoms and stabilization of the patient’s condition. This enhanced control reduces the need to switch to MMF as an alternative immunosuppressive treatment. Essentially, when patients respond well to higher doses of steroids, the clinical need to seek additional or alternative treatments like MMF diminishes. However, it is important to note that maintaining high steroid doses is not feasible for all patients, considering comorbidities and potential side effects.

Moreover, our analysis revealed a significant correlation between the delta variations in MGCS scores at 12 months and sex, indicating that the change in MGCS scores over the 12-month period differed significantly between males and females. This finding warrants further investigation to understand the underlying reasons for this sex-related difference in response to MMF.

No significant correlations were found between delta variations in MGCS or MGADL scores and other clinical variables, including serotype, clinical phenotype, thymectomy status, and MGFA classification at disease onset. This could suggest that the efficacy of MMF in improving MG symptoms is consistent across different subgroups of patients, making it a versatile treatment option for various MG phenotypes.

## 5. Limitations

This study has several limitations, including its retrospective design and relatively small sample size. Furthermore, only a minority of the sample had follow-ups at 18 and 24 months. This last factor could have influenced the non-significance of the analyses at these two time points. Moreover, this is not a direct comparative study with AZA. Thus, our conclusions regarding the effectiveness of the two immunosuppressants are based entirely on the fact that all enrolled patients had previously received AZA treatment. Finally, a steroid-sparing effect was not calculated due to a lack of data on concomitant steroid dosage at follow-up.

## 6. Conclusions

In conclusion, this study supports the efficacy and safety of MMF in the treatment of MG, with significant improvements in MGADL and MGCS scores observed from 6 months after switching from AZA. MMF demonstrated a favorable safety profile, with fewer side effects and no reported cases of infections or neoplasms. These findings suggest that MMF is an effective and well-tolerated treatment option for patients with MG, offering faster symptom control and a good safety profile. Future prospective studies with larger cohorts are needed to validate these findings and further clarify the long-term efficacy and safety of MMF in MG. Furthermore, exploring the mechanisms underlying sex-based differences in response to MMF could provide valuable insights into personalized treatment approaches for MG patients.

## Figures and Tables

**Table 1:** Summary table of demographic and clinical aspects of study population: MGFA: Myasthenia Gravis Foundation of America; AChR: acetylcholine receptor; MuSK: muscle-specific kinase; EOMG: early onset myasthenia gravis; LOMG: late onset myasthenia gravis; DSnMG: double seronegative myasthenia gravis.

Study Population	*n* = 32
Males	13 (40%)
Age (years)	66.5 ± 11.5
Age at disease onset (years)	59.4 ± 12.8
Duration of disease (months)	96.8 ± 68.5
Thymectomy	8 (25%)
MGFA at disease onset	
MGFA I	7
MGFA IIA	2
MGFA IIB	9
MGFA IIIA	8
MGFA IIIB	5
MGFA V	1
MGADL at baseline	6.4 ± 5.3
MGCS at baseline	7.6 ± 4.3
Serotype	
AChR	26 (81%)
MuSK	2 (6%)
DSnMG	4 (13%)
Type of onset	
Early onset (<60)	18 (56%)
Late onset (≥60)	14 (44%)
Treatments	
Pyridostigmine	28 (88%)
Prednisone	28 (88%)
Switch from azathioprine	32 (100%)
Switch to other drugs because of ineffectiveness	4 (12.5%)
Add-on to monocolonal antibodies	4 (12.5%)
Side effect	4 (12.5%)

## Data Availability

The original contributions presented in the study are included in the article, further inquiries can be directed to the corresponding author.
